# Superconductivity in Hourglass Dirac Chain Metals (Ti, Hf)IrGe

**DOI:** 10.1002/advs.202512434

**Published:** 2025-09-30

**Authors:** Pavan Kumar Meena, Dibyendu Samanta, Sonika Jangid, Roshan Kumar Kushwaha, Rhea Stewart, Adrian D. Hillier, Sudeep Kumar Ghosh, Ravi Prakash Singh

**Affiliations:** ^1^ Department of Physics Indian Institute of Science Education and Research Bhopal Bhopal 462066 India; ^2^ Department of Physics Indian Institute of Technology Kanpur 208016 India; ^3^ ISIS Facility STFC Rutherford Appleton Laboratory Didcot OX11 0QX UK

**Keywords:** Dirac chain metals, hourglass, topological superconductivity

## Abstract

Realizing superconductivity in stoichiometric topological materials is a major focus in condensed matter physics, as it paves the way to achieve topological superconductivity. Here, it is reported that the discovery of ternary germanide superconductors, *M*IrGe (*M* = Ti, Hf), predicted to exhibit non‐symmorphic symmetry‐protected hourglass Dirac chains, can be prime candidates for topological superconductivity. Using comprehensive thermodynamic and muon‐spin rotation/relaxation (µSR) measurements, these materials are established as conventional bulk type‐II superconductors with transition temperatures of 2.24(5) K for TiIrGe and 5.64(4) K for HfIrGe, featuring a full gap and preserved time‐reversal symmetry. First‐principles calculations reveal striking topological features in *M*IrGe, including hourglass‐shaped bulk dispersions and a Dirac chain ‐ a ring of fourfold ‐ degenerate Dirac points protected by nonsymmorphic symmetry. Each Dirac point corresponds to the neck of the hourglass dispersion, while the Dirac chain gives rise to drumhead ‐ like surface states near the Fermi level. Additionally, nontrivial $Z_{2}$ topology leads to isolated Dirac surface states with helical spin textures that disperse across the Fermi level, forming an ideal platform for proximity‐induced topological superconductivity. The coexistence of conventional bulk superconductivity, symmetry‐protected hourglass topology, and helical spin‐textured surface states suggests *M*IrGe as a rare platform to achieve topological superconductivity, opening new avenues for next‐generation quantum technologies.

## Graphical abstract

We report the discovery of ternary germanide superconductors MIrGe (M = Ti, Hf) that host hourglass Dirac chains protected by nonsymmorphic symmetry. These compounds exhibit bulk type‐II superconductivity with fully gapped states and preserved time‐reversal symmetry, as confirmed by µSR measurements. Their distinctive topology and associated drumhead surface states make them promising platforms for realizing topological superconductivity and enabling quantum device applications.

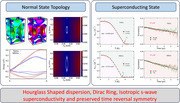

## 1 Introduction

Symmetry‐protected topological phases have revolutionized quantum materials research, leading to the discovery of Dirac and Weyl semimetals in materials like Na_3_Bi, Cd_3_As_2_, TaAs, LaAlGe, and MoTe_2_.^[^ ^1^, ^2^, ^3^ ^]^ These systems exhibit unique transport signatures and robust surface states driven by non‐trivial topology of their bulk electronic structures.^[^ ^2^, ^4^ ^]^ A key breakthrough has been the recognition of non‐symmorphic crystal symmetries, which enforce essential band crossings and entangle multiple bands, giving rise to unconventional fermionic excitations such as hourglass fermions.^[^ ^5^, ^6^ ^]^ Characterized by hourglass‐shaped bulk dispersions, these fermions are stabilized by nonsymmorphic symmetries like glide mirrors and screw axes, ensuring robust surface states across different crystal orientations.^[^ ^5^, ^6^ ^]^ Unlike topological insulators, which rely solely on time‐reversal symmetry (TRS),^[^ ^7^ ^]^ hourglass metals benefit from additional symmetry protection, making them promising candidates for advanced electronics due to their spin‐momentum locking and high spin‐Hall conductivity.^[^ ^5^, ^8^, ^9^, ^10^, ^11^ ^]^

In parallel, topological superconductors have emerged as a focal point in quantum research, particularly due to their potential in topological quantum computing.^[^ ^12^, ^13^, ^14^ ^]^ While efforts to realize topological superconductivity have often relied on proximity effects or doping in materials like Bi_2_Se_3_^[^ ^15^, ^16^, ^17^, ^18^ ^]^ and SnTe,^[^ ^19^ ^]^ these approaches face challenges such as interface effects and lattice mismatches. This has spurred the search for intrinsic topological superconductors in stoichiometric materials^[^ ^20^, ^21^ ^]^ that combine high superconducting transition temperatures (*T*_*c*_) with isolated topological surface states at the Fermi level. Although candidates like Au_2_Pb^[^ ^22^ ^]^ and PdTe_2_^[^ ^23^ ^]^ have been identified, their low *T*_*c*_ values underscore the rarity of ideal materials. Bulk superconducting topological metals, which intertwine topology, correlations, and novel superconducting ground states, offer a promising platform for realizing intrinsic topological superconductivity and exploring exotic quantum phenomena.

Equiatomic ternary silicide and germanide materials^[^ ^24^, ^25^, ^26^, ^27^ ^]^ have garnered significant interest due to their structural diversity and wide range of correlated electron phenomena, including magnetism,^[^ ^28^ ^]^ Kondo lattice behavior,^[^ ^24^ ^]^ and time‐reversal symmetry‐breaking (TRSB) unconventional superconductors.^[^ ^29^, ^30^, ^31^ ^]^ Among these, compounds crystallizing in the orthorhombic Pnma (No. 62) space group^[^ ^26^, ^32^ ^]^ have emerged as a promising platform for exploring unconventional superconductivity and symmetry‐protected topological states. Recent studies have shown that Dirac semimetallic silicides such as (Nb, Ta)OsSi,^[^ ^33^ ^]^ as well as ZrIrSi and HfIrSi, exhibit nontrivial band topologies protected by nonsymmorphic symmetries.^[^ ^34^, ^35^, ^36^ ^]^ Notably, these compounds are isostructural with the *M*IrGe (*M* = Ti, Hf) series,^[^ ^37^ ^]^ underscoring the broader relevance of this structural motif. The superconducting behavior across this family is remarkably diverse, ranging from fully gapped conventional superconductivity in ScRuSi, ZrRhSi, ZrIrSi, and HfIrSi,^[^ ^34^, ^35^, ^36^ ^]^ ferromagnetic superconductivity in U(Rh, Co)Ge^[^ ^38^ ^]^ to TRSB superconductivity in (Nb, Ta)OsSi.^[^ ^33^ ^]^ This diversity highlights the rich phenomenology of the superconducting state enabled by nonsymmorphic symmetry and strongly motivates further investigation of the relatively unexplored bulk superconductivity of *M*IrGe (*M* = Ti, Hf)^[^ ^37^ ^]^ compounds.

In this paper, we investigate the ternary germanides *M*IrGe (*M* = Ti, Hf)^[^ ^37^ ^]^ as promising candidates for topological superconductors using a combination of experimental and theoretical techniques. Substituting Ti (3*d*) with Hf (5*d*) enables exploration of the effects of enhanced spin‐orbit coupling. Resistivity, magnetization, and specific heat measurements confirm bulk conventional type‐II superconductivity, while muon‐spin rotation/relaxation (µSR) studies reveal an isotropic s‐wave superconducting gap with preserved TRS. First‐principles calculations and symmetry analysis reveal hourglass‐type bulk dispersions, where the necks form Dirac rings protected by nonsymmorphic symmetries, leading to drumhead‐like surface states. These materials also host well‐separated Dirac topological surface states with helical spin textures due to nontrivial $Z_{2}$ topology. These robust, symmetry‐protected topological surface states disperse across the Fermi level and remain distinct from the bulk states, providing an ideal platform to investigate the interplay between nonsymmorphic symmetry‐protected topological features and superconductivity.

## 2 Results

### 2.1 Structural Characterization and Bulk Superconductivity

**Figure 1:**
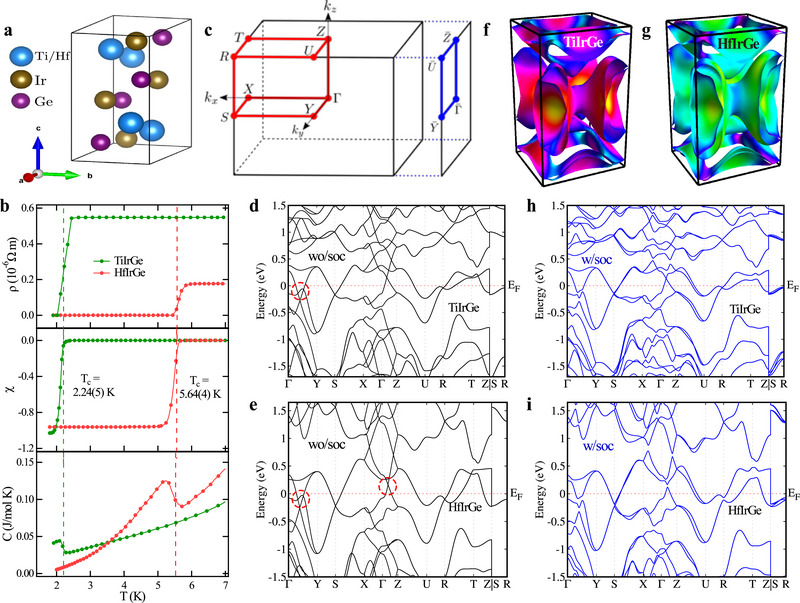
Bulk superconductivity and electronic band structure of *M*IrGe (*M* = Ti, Hf): a) Schematic of the crystal structure of *M*IrGe (*M* = Ti, Hf). b) Temperature variation of resistivity (top), magnetization (middle) in zero‐field‐cooled warming and field‐cooled cooling modes at an external magnetic field µ_0_ *H* = 1.0 mT, and specific heat (bottom), showing superconductivity of *M*IrGe (*M* = Ti, Hf). c) Bulk Brillouin zone (BZ) and its projection onto the (100) surface, with red dots and blue lines indicating high‐symmetry points and paths, respectively. d,e) Electronic band structures of TiIrGe and HfIrGe without spin‐orbit coupling (SOC). f,g) Combined Fermi surfaces with SOC for TiIrGe and HfIrGe, respectively. The band crossings that lead to glide mirror symmetry‐protected nodal rings are shown by red circles. h,i) Electronic band structures with SOC for TiIrGe and HfIrGe, respectively.

Polycrystalline samples of the ternary germanides *M*IrGe (*M* = Ti, Hf) were synthesized by the arc melting method. Powder X‐ray diffraction (XRD) measurements confirm that *M*IrGe (*M* = Ti, Hf) crystallizes in an orthorhombic TiNiSi‐type structure (**Figure** **1**a), with nonsymmorphic space group symmetry Pnma (No. 62) and the point group *D*_2*h*_ (see Supporting Information (SM)). The temperature dependence of resistivity, magnetic susceptibility, and specific heat of *M*IrGe (*M* = Ti, Hf), as shown in Figure 1b, confirms bulk superconductivity, exhibiting a sharp resistivity drop, full diamagnetic screening in zero‐field cooled warming at an applied field of 1.0 mT, and a pronounced jump in specific heat at the transition temperature (*T*_*c*_). The observed *T*_*c*_ values are approximately 2.24(5) K for TiIrGe and 5.64(4) K for HfIrGe. The normal‐state resistivity, fitted to the parallel resistor model, decreases with temperature, showing metallic behavior with RRR values of 5.14(6) for TiIrGe and 6.52(7) for HfIrGe. The FCC and ZFCW curves indicate flux pinning and type‐II superconductivity. Field‐dependent magnetization and temperature‐dependent resistivity/magnetization measurements enabled the estimation of the lower and upper critical fields by fitting the temperature dependence of these critical fields with the Ginzburg‐Landau model (GL) (see SM for details). The lower critical field values *H*_*c*1_(0) are 5.6 (1) mT for TiIrGe and 36.4 (1) mT for HfIrGe, while the upper critical fields *H*_*c*2_(0) are 0.68(1) and 1.36(1) T (from magnetization) and 0.71(1) and 2.04(1) T (from resistivity) for TiIrGe and HfIrGe, respectively. Using the *H*_*c*1_(0) and *H*_*c*2_(0) values, the obtained coherence lengths ξ_*GL*_ for TiIrGe and HfIrGe are 22.0(2) and 15.5(6) nm, respectively, while the penetration depths λ_*GL*_ are 279.1(5) and 92.6(3) nm, yielding GL parameters κ_*GL*_ of 12.6(8) and 5.9(5), with thermodynamic critical fields H_*c*_ estimated at 38.7(3) and 166.6(2) mT, indicating strong type‐II superconductivity. The low value of the Maki parameters (0.18(1) for TiIrGe and 0.15(1) for HfIrGe) suggests that orbital limiting effects dominate over the Pauli limiting effect in their superconductivity (see Supporting Information for detailed calculation). Normal state‐specific heat data are fitted to the Debye–Sommerfeld relation, the resulting Debye temperatures θ_*D*_ are 399(5) and 301(2) K, and the density of states at the Fermi level *D*_*C*_(*E*_*F*_) is 4.21(6) and 3.15(6) states *eV* ^−1^ *f*.*u*.^−1^, respectively. The electron‐phonon coupling constants λ_*e* − *ph*_, calculated from McMillan's equation, are 0.48(9) and 0.65(7) for TiIrGe and HfIrGe, suggesting weak‐coupling superconductivity. Electronic specific heat *C*_*el*_(*T*) is well fitted with the fully gapped weak‐coupling BCS model (see SM), yielding superconducting gaps of Δ/*k*_*B*_ *T*_*c*_ = 1.47(2) and 2.04(2) for TiIrGe and HfIrGe, respectively.

### 2.2 Electronic Band Structure

To elucidate the characteristics of the normal state of *M*IrGe (*M* = Ti, Hf), we performed detailed calculations of the electronic band structure calculations from first principles using density functional theory (DFT) within the generalized gradient approximation (GGA).^[^ ^39^ ^]^ The 3D bulk and (100) 2D surface Brillouin zones of *M*IrGe (*M* = Ti, Hf) are schematically shown in Figure 1c. Calculated electronic band structures without the effects of spin‐orbit coupling (SOC) are shown in Figure 1d,e for TiIrGe and HfIrGe, respectively. We note that multiple dispersive bands intersect the Fermi level, forming various electron and hole pockets, indicating the multi‐band nature of *M*IrGe (*M* = Ti and Hf). These two isostructural compounds show similar band dispersions, featuring band crossing points along the Γ − *Y* and Γ − *Z* paths in the Brillouin zone, indicated by red circles. The low‐energy bands near the Fermi level in TiIrGe are predominantly composed of Ti‐3d, Ir‐5d, Ge‐4p, and Ir‐5p orbitals, whereas in HfIrGe, they consist of Hf‐5d, Ir‐5d, Ge‐4p, Ir‐5p, and Hf‐5p orbitals, both in descending order of significance. These compositions align with the calculated orbital‐resolved projected density of states (DOS) (see Supporting Information for details). SOC induces significant splittings in the band structures near the Fermi level, as shown in Figure 1d,e for TiIrGe and HfIrGe, respectively, with SOC. The estimated maximum band splittings caused by SOC are along the *RT*‐direction and are given by ∼100 meV for TiIrGe and ∼150 meV for HfIrGe, reflecting the substantial contributions to the bands from the Ti 3*d* and Hf 5*d* orbitals, respectively. The SOC band structures of both compounds reveal distinctive hourglass‐type dispersions along the S‐X and S‐R high‐symmetry directions, for example, as illustrated in detail later. The combined Fermi surface with parallel sheets of TiIrGe and HfIrGe with SOC is shown in Figure 1f,g, respectively. We note that there are several parallel Fermi surface sheets spread across the Brillouin zone, raising the possibility of dominant interband superconducting pairing.^[^ ^33^, ^40^ ^]^

### 2.3 Muon Spin Rotation and Relaxation (µSR) Results

Comprehensive µSR measurements were conducted using the MuSR spectrometer at the ISIS Pulsed Neutron and Muon Source in the United Kingdom.^[^ ^41^ ^]^ The µSR technique, renowned for its exceptional sensitivity arising from the muons significant magnetic moment and large gyromagnetic ratio, provides invaluable insights into the ground‐state properties of superconductors. Results from the transverse‐field (TF) and longitudinal‐field (LF) configurations are discussed below.

#### 2.3.1 TF‐µSR

**Figure 2:**
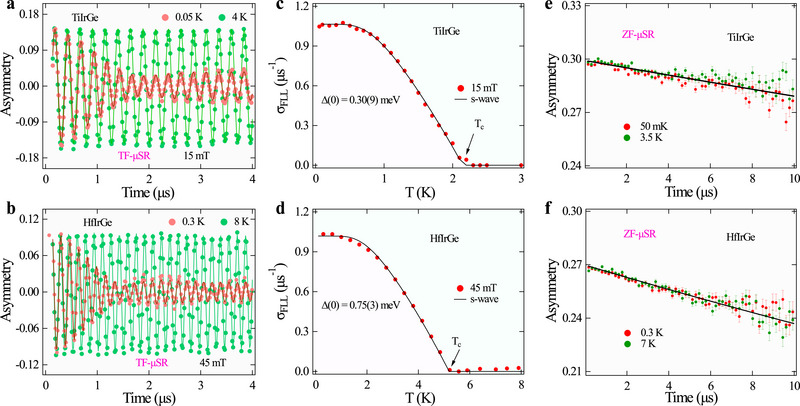
Microscopic muon spin rotation and relaxation (µSR) results of *M*IrGe (*M* = Ti, Hf): a,b) show TF‐µSR asymmetry spectra at 15 and 45 mT for Ti and Hf‐based compounds in the superconducting and normal states, with solid lines representing the fit using eq. 1. c,d) Temperature‐dependent relaxation rate fitted with an s‐wave model in the superconducting states for both samples. e,f) display ZF‐µSR asymmetry spectra below and above *T*_*c*_, with solid lines representing the respective fit for TiIrGe and HfIrGe.

TF‐µSR measurements, with an external magnetic field applied perpendicular to the muon spin polarization, probe the superconducting gap structure, revealing insights into carrier density in the superconducting state through the formation of a flux line lattice (FLL) in fields between the lower (*H*_*c*1_) and upper (*H*_*c*2_) critical fields. Asymmetry spectra were recorded at temperatures from 50 mK to 4 K and 0.3 to8 K for the TiIrGe and HfIrGe samples under different applied transverse magnetic fields. **Figure** **2**a,b show representative spectra for the TiIrGe and HfIrGe samples above and below *T*_*c*_ in fields of 15 and 45 mT, respectively. Above *T*_*c*_, there is a small normal state relaxation due to randomly oriented nuclear moments, whereas below *T*_*c*_, the formation of the FLL in the mixed state results in an inhomogeneous magnetic field that causes a dramatic increase in damping. The time evolution of the TF asymmetry can be well described by the Gaussian‐damped oscillatory function given as ref. [42, 43]:

(1) $$A\left(t\right)=A_{1}e^{-\sigma^{2}t^{2}/2}\cos\left(\gamma_{\mu }B_{1}t+\phi \right)+A_{2}\cos\left(\gamma_{\mu }B_{2}t+\phi \right)$$

where *A*_1_ and *A*_2_ denote the initial asymmetries of the sample and the non‐relaxing background from the silver sample holder, respectively, while the corresponding local magnetic fields sensed by the muons are *B*_1_ and *B*_2_.γ_µ_/2π = 135.5 MHz/T is the muon gyromagnetic ratio, and ϕ is the phase of the initial muon spin polarization with respect to the detector. Above *T*_*c*_, the internal magnetic field equals the applied field, while in the superconducting state, the Meissner effect reduces the internal field (see Supporting Information). The superconducting relaxation rate (σ_*FLL*_) is calculated using $\sigma_{FLL}=\sqrt{\sigma^{2}-\sigma_{N}^{2}}$, where σ is the total relaxation rate, while σ_*N*_ accounts for the temperature‐independent normal state relaxation due to the randomly oriented nuclear spins. The σ_*N*_ values are 0.049(2) and 0.033(6) µ*s* ^−1^ for the TiIrGe and HfIrGe compounds, respectively. The temperature‐dependent relaxation σ_*FLL*_(*T*), directly proportional to the penetration depth and superconducting fluid density, is fitted with the equation:

(2) $$\frac{\sigma_{FLL}\left(T\right)}{\sigma_{FLL}\left(0\right)}=\frac{\lambda^{-2}\left(T\right)}{\lambda^{-2}\left(0\right)}=1+2\int_{\Delta (T)}^{\infty }\left(\frac{\partial f}{\partial E}\right)\frac{EdE}{\sqrt{E^{2}-\Delta {\left(T\right)}^{2}}}$$

where λ(0) is the London penetration depth, $f={\left[1+e^{\left(E/k_{B}T\right)}\right]}^{-1}$ is the Fermi distribution function and Δ(*T*) is the BCS superconducting gap function, defined by Δ(*T*) = Δ_0_tanh [1.82(1.018((*T*_*c*_/*T*) − 1))^0.51^].^[^ ^44^ ^]^ The σ_*FLL*_(*T*) were well described by the isotropic BCS s‐wave model (Figure 2c,d), providing superconducting gaps of Δ(0) = 0.30(9) and 0.75(2) meV, with normalized gaps Δ(0)/*k*_*B*_ *T*_*C*_ = 1.66(7) and 1.68(2) for TiIrGe and HfIrGe, respectively.^[^ ^33^, ^35^, ^36^, ^45^ ^]^ Brandt has reported that, for a superconductor in the case of a low magnetic field such as *H* < <*H*_*c*2_, the field‐dependent relaxation rate σ_*sc*_ (see SM), associated with penetration depth λ(*T*), can be fitted with the equation given below:

(3) $$\sigma_{sc}\left(\mu s^{-1}\right)=4.854\times 10^{4}\left(1-h\right)\left[1+1.21{\left(1-\sqrt{h}\right)}^{3}\right]\lambda^{-2}$$

with *h* = *H*/*H*_*c*2_(0) the reduced field. Obtained London penetration depth λ^µ*SR*^(0) values are 2731(6) and 2461(9) Å for TiIrGe and HfIrGe, respectively. These values are comparable to the other structurally similar compounds (Zr, Hf)IrSi.^[^ ^33^, ^35^, ^36^, ^45^ ^]^ Notably, the λ^µ*SR*^(0) value for TiIrGe aligns closely with magnetization estimates, while HfIrGe shows a significant discrepancy. This divergence, observed in other compounds as well, may reflect a genuine difference in determining penetration depth from the lower critical field (in the Meissner state) and superfluid density (in the vortex state).^[^ ^46^, ^47^, ^48^ ^]^ The SM provides the electronic properties and includes the Uemura plot.

#### 2.3.2 ZF‐µSR

ZF‐µSR is used to detect the precession of muon spins in local magnetic fields in the sample, which can reveal the presence or absence of time‐reversal symmetry in the superconducting state. ZF‐µSR asymmetry spectra for polycrystalline *M*IrGe (*M* = Ti and Hf) are similar above and below T_*c*_ as shown in Figure 2e,f, ruling out time‐reversal symmetry breaking (TRSB) due to the presence of a spontaneous magnetic field within the detection limits (up to 1 µT). A similar trend with a lower asymmetry value is also reported in silicides.^[^ ^36^, ^49^ ^]^ The absence of coherent oscillations or fast decays in the spectra indicates that there is no magnetic order or fluctuations, despite the possibility of TRSB‐induced magnetization increasing relaxation rates.^[^ ^50^ ^]^ In non‐magnetic materials, the depolarization of muon spins in zero field is mainly determined by the randomly oriented nuclear magnetic moments. The ZF depolarization data was fitted with a Lorentzian function:

(4) $$G_{ZF}\left(t\right)=A_{0}\left(t\right)\exp\left(-\lambda t\right)+A_{bg}$$

where *A*_0_ is the initial asymmetry corresponding to the sample, and *A*_*bg*_ considers the background asymmetry associated with muon stopping in the silver sample holder, which is nearly temperature‐independent. The relaxation rate λ, associated with nuclear moments, shows negligible temperature dependence in the ZF‐µSR asymmetry data, suggesting that there is no evidence of TRSB in the *M*IrGe samples. However, the breaking of TRS observed in other isostructural compounds^[^ ^33^ ^]^ raises questions about the role of spin‐orbit coupling in these materials. Further theoretical and experimental work is required to understand the presence or absence of TRSB in superconducting materials with topological characteristics. It will help us to understand the pairing mechanisms and experimental signatures of topological superconductivity, which are still lacking.

### 2.4 Topology of Electronic Band Structure

Both *M*IrGe (*M* = Ti, Hf) have rich topological features in their electronic band structures that are protected by nonsymmorphic glide mirror symmetries. The complete symmetry of the crystal structure of *M*IrGe (*M* = Ti and Hf) can be generated by the three key symmetry operations: inversion symmetry P:(x,y,z)→(−x,−y,z), mirror symmetry $M_{y}:\left(x,y,z\right)\to \left(x,-y+\frac{1}{2},z\right)$, and glide mirror symmetry $G_{x}:\left(x,y,z\right)\to \left(-x+\frac{1}{2},y+\frac{1}{2},z+\frac{1}{2}\right)$. The two isostructural compounds show similar band dispersions without SOC, featuring band crossing points along the Γ − *Y* and Γ − *Z* paths in the Brillouin zone, as indicated by the red circles in Figure 1d,e. These crossings give rise to a nodal ring encircling the Γ point in the *k*_*x*_ = 0 plane (see Supporting Information for details), protected by the glide mirror symmetry $G_{x}$. When SOC is included (Figure 1h,i), the nodal ring surrounding the Γ point in the *k*_*x*_ = 0 plane fully gaps out, leading to band inversion near the Γ point in both compounds and consequently nontrivial topology. Although the *M*IrGe compounds (*M* = Ti, Hf) do not have a global bandgap across the entire Brillouin zone (BZ) with SOC, we can still define the topological invariant $Z_{2}$ in the *k*_*y*_ = 0 plane. Our calculations reveal that *M*IrGe has a topological index of $Z_{2}=1$, indicating the presence of nontrivial topological surface states on the (010) surface that have a helical spin texture, are well separated from the bulk, and disperse across the Fermi level (see Supporting Information for details).

**Figure 3:**
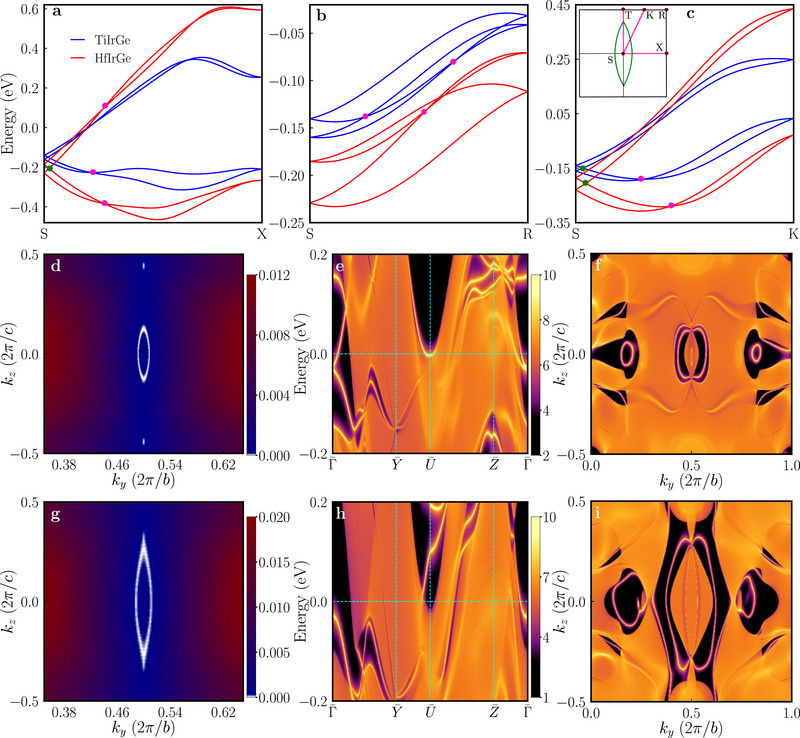
Hourglass dispersions, Dirac rings and surface states of *M*IrGe (*M* = Ti, Hf) with SOC: a–c) Hourglass‐type band dispersions for TiIrGe and HfIrGe along the high‐symmetry directions *S* − *X*, *S* − *R* and *S* − *K* (TiIrGe in blue, HfIrGe in red). Here, *K* represents the midpoint between *T* and *R* as shown in the inset of (c). Type‐I and type‐II Dirac fermions are marked by green and pink dots, respectively. The inset depicts the schematic of the fourfold degenerate Dirac ring formed by neck points (green dots) of the hybrid hourglass dispersion on the *k*_*x*_ = π plane. d,g) Distribution of the hourglass Dirac ring (white) surrounding point S for TiIrGe and HfIrGe, respectively. The color scale indicates the local gap between crossing bands. e,h) Surface state spectrum along the high‐symmetry paths in the projected (100) 2D surface Brillouin zone for TiIrGe and HfIrGe, respectively. f,i) Surface Fermi arcs (constant energy slice of the spectrum) for TiIrGe at energy –0.138 eV and for HfIrGe at energy –0.130 eV, respectively.

The band structures of both compounds with SOC reveal distinctive hourglass‐type dispersions along the *S* − *X*, *S* − *R* and *S* − *K* (where *K* denotes the midpoint between *T* and *R*) high‐symmetry paths, as shown in Figure 1h,i. Further analysis reveals several distinct topological features: glide mirror $G_{x}$ protected hourglass dispersions, a Dirac ring around the S‐point in the *k*_*x*_ = π plane, and nontrivial topological surface states. The zoomed versions of the unique hourglass‐shaped dispersions near the Fermi levels protected by the glide mirror symmetry $G_{x}$ along the *S* − *X*, *S* − *R*, and *S* − *K* high‐symmetry paths are shown in **Figure** **3**a–c.

First we examine the hourglass dispersion along the *S* − *R* high symmetry path [i.e. along (π, π, *k*_*z*_), where −π < *k*_*z*_ < π]. This path in the *k*_*x*_ = π plane remains invariant under the glide mirror operation $G_{x}$. Along *S* − *R*, $G_{x}^{2}=T_{011}\bar{E}=e^{-ik_{z}}$, where $\bar{E}$ denotes a 2π spin rotation and $T_{011}$ translates (*x*, *y*, *z*) to (*x*, *y* + *b*, *z* + *c*). Consequently, the eigenvalue *g*_*x*_ of $G_{x}$ must be $\pm e^{-ik_{z}/2}$. For a Bloch state $|\phi_{n}\rangle$, its Kramer's partner $PT|\phi_{n}\rangle$ satisfies $G_{x}\left(\mathrm{PT}|\phi_{n}\rangle \right)=g_{x}\left(\mathrm{PT}|\phi_{n}\rangle \right)$, sharing the same eigenvalue *g*_*x*_. At the time‐reversal invariant momenta (TRIM) points *S* and *R*, $G_{x}\left(T|\phi_{n}\rangle \right)=g_{x}\left(T|\phi_{n}\rangle \right)$. Given the centrosymmetric crystal structures of *M*IrGe (*M* = Ti, Hf), $G_{x}\left(P|\phi_{n}\rangle \right)=g_{x}\left(P|\phi_{n}\rangle \right)$. At *S* (π, π, 0), the states $|\phi_{n}\rangle$, $T|\phi_{n}\rangle$, $P|\phi_{n}\rangle$, and $\mathrm{PT}|\phi_{n}\rangle$ then form a degenerate quartet with *g*_*x*_ = ±1 (*g*_*x*_ = −1 has a lower energy than *g*_*x*_ = +1). At *R* (π, π, π), *g*_*x*_ = ±*i*, where the Kramer's partners have opposite eigenvalues, forming a quartet with two states with *g*_*x*_ = +*i* and the other two with *g*_*x*_ = −*i*. This symmetry‐enforced eigenvalue redistribution between the points *S* and *R* necessarily leads to the symmetry‐protected band crossing, manifesting as the characteristic hourglass dispersion shown in Figure 3b. Since these band crossings are enforced by nonsymmorphic glide mirror symmetry and independence from band inversion mechanism, they are very robust. Similarly, topological analysis also applies to the *S* − *X* and *S* − *K* high‐symmetry paths leading analogously to glide mirror symmetry‐protected hourglass dispersions as shown in Figure 3a,c. We note that the hourglass dispersions are spread across a very large energy window crossing the Fermi level along the *S* − *X* and *S* − *K* directions (Figure 3a,c) in contrast to the dispersions along the *S* − *R* direction in a very narrow energy window below the Fermi level (Figure 3b).

Figure 3a–c also feature additional type‐I and type‐II Dirac point crossings shown by the green and pink dots, respectively. Type‐I Dirac points (neck points) arise for any *S* − *K* path, where *K* is an arbitrary point along the *T* − *R* direction. A similar concept also applies to the *R* − *X* high symmetry path. Consequently, the neck points (green dots) of the hourglass‐type dispersion in *M*IrGe (*M* = Ti and Hf) are guaranteed and form a continuous Dirac nodal ring around the point *S* in the *k*_*x*_ = π plane (as schematically shown in the inset of Figure 3c). This hybrid hourglass‐type Dirac ring, confirmed by DFT calculations and shown for TiIrGe and HfIrGe in Figure 3d,g, respectively, is fundamentally determined by the nonsymmorphic space group symmetry.

Surface electronic structure analysis of TiIrGe and HfIrGe, projected onto the (100) surface Brillouin zones (Figure 3e,f), unveils multiple topologically non‐trivial surface states intersecting the Fermi level. These materials exhibit bulk nodal loops that manifest as characteristic drumhead‐like surface states in regions where the loops project finite areas onto the surface.^[^ ^10^ ^]^ Due to broken inversion symmetry on the surface, the drumhead surface bands show splitting.^[^ ^10^ ^]^ The states, which are fundamentally connected to the bulk Dirac rings, emerge at specific energies: −0.138 eV in TiIrGe and −0.130 eV in HfIrGe (Figure 3h,i).

## 3 Discussion

The low symmetry crystal structure of *M*IrGe (*M* = Ti and Hf) and the strong effects of SOC on their electronic band structure provide a unique opportunity to determine the symmetry of the superconducting order parameter. Using a Ginzburg–Landau theory‐based symmetry analysis^[^ ^50^ ^]^ of the superconducting order parameters in the strong SOC limit within an effective single‐band picture corresponding to the point group *D*_2*h*_, we find that all the symmetry allowed superconducting order parameters apart from the one in the fully symmetric ^1^ *A*_1_‐channel have nodes.^[^ ^51^ ^]^ Nonsymmorphic symmetries can also lead to additional symmetry‐enforced nodes at the Brillouin Zone boundaries or zone faces.^[^ ^52^ ^]^ Since *M*IrGe (*M* = Ti and Hf) shows bulk superconductivity with a single gap and no signatures of TRS breaking, the leading superconducting instability in these materials will be in the ^1^ *A*_1_ s‐wave singlet channel coming mostly from phonon‐mediated on‐site pairing. The inherently multiband character of *M*IrGe leading to a pair of parallel Fermi surface sheets, which are close to each other throughout the Brillouin zone as shown in Figure 1f,g, will give rise to a strong interband pairing. Further theoretical investigations using a detailed tight‐binding model of the band structure of *M*IrGe, for example, with on‐site singlet pairing, are necessary to calculate the novel transport properties^[^ ^11^ ^]^ expected in these materials.

We computed the phonon dispersions of *M*IrGe (*M* = Ti, Hf) that reveal real positive phonon spectra throughout the entire Brillouin zone, confirming the dynamic stability of their crystal structures. Using phonon spectra, the superconducting transition temperature (*T*_*c*_) is calculated using the McMillan formula,^[^ ^53^ ^]^ assuming phonon‐mediated s‐wave superconductivity in these materials. *T*_*c*_ is determined by evaluating electron–phonon coupling matrix elements through the Eliashberg spectral function α^2^ *F*(ω).^[^ ^54^, ^55^ ^]^ The cumulative electron‐phonon coupling constant (λ) is given by $\lambda =2\int \frac{\alpha^{2}F\left(\omega \right)}{\omega }d\omega$. Using these parameters, the superconducting critical temperature *T*_*c*_ is calculated using the modified McMillan formula:^[^ ^53^ ^]^ $T_{c}=\frac{\omega_{\log}}{1.2}\exp\left[\frac{-1.04(1+\lambda )}{\lambda -\mu^{*}\left(1+0.62\lambda \right)}\right]$. µ* is an empirical parameter that describes the screened Coulomb interaction^[^ ^56^ ^]^ and has typical values between 0.1 and 0.16. The logarithmically averaged phonon frequency is $\omega_{\log}=\exp\left[\frac{2}{\lambda }\int \frac{\alpha^{2}F\left(\omega \right)}{\omega }\log\left(\omega \right)d\omega \right]$. We obtained *T*_*c*_ for TiIrGe and HfIrGe to be 2.71 K and 5.08 K, respectively, assuming µ* = 0.10,^[^ ^56^ ^]^ in close agreement with the experimental results.

The symmetry‐protected topological surface states in *M*IrGe (*M* = Ti and Hf) are well separated from the bulk states and dispersed across the Fermi level, enabling the emergence of a distinct surface superconducting gap through proximity coupling with the bulk superconducting condensate. This interplay establishes *M*IrGe as a promising platform to realize surface topological superconductivity. The coexistence of a distinct bulk superconducting gap and a smaller surface gap can be experimentally probed using techniques such as Andreev reflection spectroscopy, as demonstrated in YRuB_2_,^[^ ^57^ ^]^ or angle‐resolved photoemission spectroscopy (ARPES), as applied to PdTe.^[^ ^58^ ^]^ Moreover, systems with nonsymmorphic space group symmetry constraints can realize symmetry‐enforced nodal or fully gapped bulk topological superconducting states, depending on the pairing symmetry realized, even in the absence of strong spin‐orbit coupling.^[^ ^52^, ^59^, ^60^ ^]^

## 4 Summary and Conclusion

We systematically uncover the superconducting and topological properties of polycrystalline *M*IrGe (*M* = Ti and Hf), demonstrating a striking interplay between conventional superconductivity and nonsymmorphic symmetry‐protected hourglass topology, thereby establishing these materials as robust stoichiometric platforms for realizing topological superconductivity. X‐ray diffraction confirms the TiNiSi‐type orthorhombic crystal structure with nonsymmorphic space group symmetry. Bulk superconductivity is established through resistivity, magnetization, and specific heat measurements, yielding *T*_*c*_ ≈ 2.24(5) K for TiIrGe and 5.64(4) K for HfIrGe. Critical field and specific heat analysis indicate conventional type‐II weak‐coupling BCS superconductivity with an isotropic s‐wave gap, further supported by muon spin rotation and relaxation (µSR) measurements confirming fully gapped superconductivity with preserved time‐reversal symmetry. Our first‐principles calculations on *M*IrGe (*M* = Ti and Hf) predict a rich topological landscape, featuring nonsymmorphic glide mirror symmetry‐protected hybrid hourglass bulk dispersions, where the necks of these dispersions form a Dirac chain that generates drumhead‐like surface states. Notably, topological surface states arising from band inversion around the Γ‐point cross the Fermi level with a helical spin texture on the (100) surface, providing an ideal setting to explore topological superconductivity. The predicted topological features in *M*IrGe can be experimentally verified, for example, using ARPES or scanning tunneling microscopy/spectroscopy (STM/STS).^[^ ^61^ ^]^ However, these measurements require high‐quality single‐crystal surfaces of MIrGe compounds, which are currently unavailable despite repeated synthesis efforts and constitute an important direction for future research.

As nonsymmorphic symmetry‐protected hourglass Dirac chain metals that are also superconductors, *M*IrGe compounds offer exciting prospects for advanced quantum technologies. Their symmetry‐protected topological surface states can enable potential platforms for proximity‐induced surface topological superconductivity,^[^ ^10^ ^]^ possibly hosting robust Majorana modes for fault‐tolerant quantum computing. The coexistence of helical spin textures and superconductivity could facilitate spin‐polarized supercurrents, essential for spintronic applications.^[^ ^11^, ^62^ ^]^ Bulk Dirac nodal loops in *M*IrGe will lead to emergent unconventional low‐energy excitations, which can give rise to novel superconducting instabilities with unconventional pairing mechanisms. Additionally, these materials can form Josephson junctions with unique current‐phase relationships,^[^ ^63^ ^]^ suitable for quantum interference devices, while coupling with dissipative elements could open pathways in non‐Hermitian quantum physics.^[^ ^64^, ^65^ ^]^ Their Dirac nodal loops with drumhead‐like surface states could also enable intriguing transport phenomena,^[^ ^66^, ^67^, ^68^ ^]^ including anisotropic charge transport,^[^ ^67^ ^]^ anomalous Landau levels,^[^ ^69^ ^]^ and exotic optical responses,^[^ ^70^, ^71^ ^]^ making them promising candidates for next‐generation quantum and nanoelectronic devices.

## 5 Experimental Section

### Sample Characterization

Polycrystalline *M*IrGe (*M* = Ti and Hf) samples were synthesized by arc melting of high‐purity (4*N*) Ti or Hf, Ir, and Ge in a 1:1:1 stoichiometric ratio in an Ar atmosphere, followed by rapid cooling and multiple remelts for homogeneity. The ingots were then annealed at 850 °C for 7 days. Phase purity and crystal structure were analyzed by powder X‐ray diffraction (XRD) with Cu *K*_α_ radiation (λ = 1.5406 Å) on a PANalytical diffractometer. Low‐temperature magnetization measurements were performed using a Quantum Design MPMS‐3 (7T) in vibrating sample magnetometry mode. Electrical and specific heat measurements were performed with the Quantum Design PPMS 9T system using four‐probe and two‐tau techniques.

### µSR Measurements

The µSR measurements were conducted on the MuSR spectrometer at the ISIS pulsed neutron and muon facility, STFC Rutherford Appleton Laboratory, United Kingdom, using 64 detectors in transverse field (TF) and zero field (ZF) configurations to investigate superconducting pairing mechanisms and spontaneous internal magnetic fields. Powder samples were mounted on a silver holder with diluted GE varnish and cooled in a dilution refrigerator. The muon asymmetry signal is determined from: *A*(*t*) = [*N*_*F*_(*t*) − α*N*_*B*_(*t*)]/[*N*_*F*_(*t*) + α*N*_*B*_(*t*)], where *N*_*B*_(*t*) and *N*_*F*_(*t*) are the number of counts in the backward and forward detector, respectively, and α is an experiment‐specific constant determined from calibration measurements taken with a small applied transverse magnetic field. The reference provides an in‐depth explanation of the technique.^[^ ^41^ ^]^ Data were analyzed using MANTID software.

### Electronic Band Structure Calculations

We performed first‐principles electronic structure calculations using density functional theory (DFT) as implemented in the QUANTUM ESPRESSO package. The Perdew‐Burke‐Ernzerhof (PBE) functional was employed within the generalized gradient approximation (GGA) to account for exchange‐correlation effects. Projector augmented wave (PAW) pseudopotentials described the electron‐ion interactions, with a plane‐wave basis set expanded to a kinetic energy cut‐off of 80 Ry. Brillouin zone integration utilized a Γ‐centered 8 × 10 × 8 Monkhorst‐Pack k‐point mesh for bulk calculations. Experimental lattice parameters and atomic positions, derived from the Rietveld refinement of X‐ray diffraction data, were used in our calculations. We constructed a tight‐binding Hamiltonian based on Maximally Localized Wannier Functions (MLWFs) using the WANNIER90 package. This Wannier‐based Hamiltonian served as input for subsequent calculations of topological properties, including surface states and nodal loops, which were performed using the WANNIER TOOLS package.

## Conflict of Interest

The authors declare no conflict of interest.

## Supporting information



Supporting Information

## Data Availability

The data that support the findings of this study are available from the corresponding author upon reasonable request.

## References

[1] N. P. Armitage , E. J. Mele , A. Vishwanath , Rev. Mod. Phys. 2018, 90, 015001.

[2] N. Ong , S. Liang , Nat. Rev. Phys. 2021, 3, 394.

[3] B. Q. Lv , T. Qian , H. Ding , Rev. Mod. Phys. 2021, 93, 025002.

[4] S. Wang , B.‐C. Lin , A.‐Q. Wang , D.‐P. Yu , Z.‐M. Liao , Adv. Phys. X 2017, 2, 518.

[5] Z. Wang , A. Alexandradinata , R. J. Cava , B. A. Bernevig , Nature 2016, 532, 189. 27075096 10.1038/nature17410

[6] A. Alexandradinata , Z. Wang , B. A. Bernevig , Phys. Rev. X 2016, 6, 021008.

[7] M. Z. Hasan , C. L. Kane , Rev. Mod. Phys. 2010, 82, 3045.

[8] B. Singh , B. Ghosh , C. Su , H. Lin , A. Agarwal , A. Bansil , Phys. Rev. Lett. 2018, 121, 226401. 30547639 10.1103/PhysRevLett.121.226401

[9] Y. Gao , P. J. Guo , K. Liu , Z. Y. Lu , Phys. Rev. B 2020, 102, 115137.

[10] S. Li , Y. Liu , S.‐S. Wang , Z.‐M. Yu , S. Guan , X.‐L. Sheng , Y. Yao , S. A. Yang , Phys. Rev. B 2018, 97, 045131.

[11] J. Ma , C. Yi , B. Lv , Z. Wang , S. Nie , L. Wang , L. Kong , Y. Huang , P. Richard , P. Zhang , K. Yaji , K. Kuroda , S. Shin , H. Weng , B. A. Bernevig , Y. Shi , T. Qian , H. Ding , Sci. Adv. 2017, 3, e1602415. 28508059 10.1126/sciadv.1602415 PMC5419706

[12] X. L. Qi , S. C. Zhang , Rev. Mod. Phys. 2011, 83, 1057.

[13] C. Nayak , S. H. Simon , A. Stern , M. Freedman , S. Das Sarma , Rev. Mod. Phys. 2008, 80, 1083.

[14] M. Sato , Y. Ando , Rep. Prog. Phys. 2017, 80, 076501. 28367833 10.1088/1361-6633/aa6ac7

[15] M. Wang , C. Liu , J. Xu , F. Yang , L. Miao , M. Yao , C. Gao , C. Shen , X. Ma , X. Chen , Z.‐A. Xu , Y. Liu , S.‐C. Zhang , D. Qian , J.‐F. Jia , Q.‐K. Xue , Science 2012, 336, 52. 22422860 10.1126/science.1216466

[16] E. Wang , H. Ding , A. V. Fedorov , W. Yao , Z. Li , Y. Lv , K. Zhao , L. Zhang , Z. Xu , J. Schneeloch , R. Zhong , S.‐H. Ji , L. Wang , K. He , X. Ma , G. Gu , H. Yao , Q.‐K. Xue , X. Chen , S. Zhou , Nat. Phys. 2013, 9, 621.

[17] M. Kriener , K. Segawa , Z. Ren , S. Sasaki , Y. Ando , Phys. Rev. Lett. 2011, 106, 127004. 21517345 10.1103/PhysRevLett.106.127004

[18] S. Sasaki , M. Kriener , K. Segawa , K. Yada , Y. Tanaka , M. Sato , Y. Ando , Phys. Rev. Lett. 2011, 107, 217001. 22181913 10.1103/PhysRevLett.107.217001

[19] S. Sasaki , Z. Ren , A. Taskin , K. Segawa , L. Fu , Y. Ando , Phys. Rev. Lett. 2012, 109, 217004. 23215610 10.1103/PhysRevLett.109.217004

[20] B. Yan , M. Jansen , C. Felser , Nat. Phys. 2013, 9, 709.

[21] S.‐Y. Guan , P.‐J. Chen , M.‐W. Chu , R. Sankar , F. Chou , H.‐T. Jeng , C.‐S. Chang , T.‐M. Chuang , Sci. Adv. 2016, 2, e1600894. 28138520 10.1126/sciadv.1600894 PMC5262470

[22] Y. Xing , H. Wang , C. Li , X. Zhang , J. Liu , Y. Zhang , J. Luo , Z. Wang , Y. Wang , L. Ling , M. Tian , S. Jia , J. Feng , X.‐J. Liu , J. Wei , J. Wang , npj Quant. Mater. 2016, 1, 1.

[23] H. Noh , J. Jeong , E. Cho , K. Kim , B. Min , B. Park , Phys. Rev. Lett. 2017, 119, 016401. 28731733 10.1103/PhysRevLett.119.016401

[24] S. Gupta , K. G. Suresh , J. Alloys Compd. 2015, 618, 562.

[25] A. V. Morozkin , Y. D. Seropegin , I. A. Sviridov , I. G. Riabinkin , J. Alloys Compd. 1999, 282, L4.

[26] G. A. Landrum , R. Hoffmann , J. Evers , H. Boysen , Inorg. Chem. 1998, 37, 5754.

[27] G. V. Subba Rao , K. Wagner , G. Balakrishnan , J. Janaki , W. Paulus , R. Schöllhorn , V. S. Subramanian , U. Poppe , Bull. Mater. Sci. 1985, 7, 215.

[28] R. Welter , G. Venturini , B. Malaman , E. Ressouche , J. Alloys Compd. 1993, 202, 165.

[29] T. Shang , S. K. Ghosh , M. Smidman , D. J. Gawryluk , C. Baines , A. Wang , W. Xie , Y. Chen , M. O. Ajeesh , M. Nicklas , E. Pomjakushina , M. Medarde , M. Shi , J. F. Annett , H. Yuan , J. Quintanilla , T. Shiroka , npj Quantum Mater. 2022, 7, 35.

[30] S. K. P., D. Singh , P. K. Biswas , A. D. Hillier , R. P. Singh , Phys. Rev. B 2018, 98, 214505.

[31] S. K. P., D. Singh , A. D. Hillier , R. P. Singh , Phys. Rev. B 2020, 102, 094515.

[32] R. Müller , R. Shelton , J. Richardson Jr , R. Jacobson , J. Less‐Common Met. 1983, 92, 177.

[33] S. K. Ghosh , P. K. Biswas , C. Xu , B. Li , J. Z. Zhao , A. D. Hillier , X. Xu , Phys. Rev. Res. 2022, 4, L012031.

[34] H. Y. Uzunok , S. Baǧcı , E. c. v. Karaca , H. M. Tütüncü , G. P. Srivastava , Phys. Rev. B 2020, 102, 134508.

[35] K. Panda , A. Bhattacharyya , P. N. Ferreira , R. Mondal , A. Thamizhavel , D. T. Adroja , C. Heil , L. T. F. Eleno , A. D. Hillier , Phys. Rev. B 2024, 109, 224517.

[36] K. Panda , A. Bhattacharyya , D. T. Adroja , N. Kase , P. K. Biswas , S. Saha , T. Das , M. R. Lees , A. D. Hillier , Phys. Rev. B 2019, 99, 174513. 10.1088/1361-648X/ab549e 31689696

[37] X. Z. Wang , B. Chevalier , J. Etourneau , P. Hagenmuller , Mater. Res. Bull. 1987, 22, 331.

[38] T. Hattori , Y. Ihara , Y. Nakai , K. Ishida , Y. Tada , S. Fujimoto , N. Kawakami , E. Osaki , K. Deguchi , N. K. Sato , I. Satoh , Phys. Rev. Lett. 2012, 108, 066403. 22401093 10.1103/PhysRevLett.108.066403

[39] J. P. Perdew , K. Burke , M. Ernzerhof , Phys. Rev. Lett. 1996, 77, 3865. 10062328 10.1103/PhysRevLett.77.3865

[40] Z. F. Weng , J. L. Zhang , M. Smidman , T. Shang , J. Quintanilla , J. F. Annett , M. Nicklas , G. M. Pang , L. Jiao , W. B. Jiang , Y. Chen , F. Steglich , H. Q. Yuan , Phys. Rev. Lett. 2016, 117, 027001. 27447519 10.1103/PhysRevLett.117.027001

[41] A. D. Hillier , S. J. Blundell , I. McKenzie , I. Umegaki , L. Shu , J. A. Wright , T. Prokscha , F. Bert , K. Shimomura , A. Berlie , H. Alberto , I. Watanabe , Nat. Rev. Methods Primers 2022, 2, 4.

[42] M. Weber , A. Amato , F. N. Gygax , A. Schenck , H. Maletta , V. N. Duginov , V. G. Grebinnik , A. B. Lazarev , V. G. Olshevsky , V. Y. Pomjakushin , V. G. Storchak , S. Kapusta , J. Bock , Phys. Rev. B 1993, 48, 13022. 10.1103/physrevb.48.13022 10007679

[43] A. Maisuradze , R. Khasanov , A. Shengelaya , H. Keller , J. Phys.: Condens. Matter 2009, 21, 075701. 21817334 10.1088/0953-8984/21/7/075701

[44] R. Prozorov , R. W. Giannetta , Supercond. Sci. Technol. 2006, 19, R41.

[45] A. Bhattacharyya , K. Panda , D. T. Adroja , N. Kase , P. K. Biswas , S. Saha , T. Das , M. R. Lees , A. D. Hillier , J. Phys.: Condens. Matter 2020, 32, 085601. 31689696 10.1088/1361-648X/ab549e

[46] V. Anand , D. Adroja , M. R. Lees , P. Biswas , A. Hillier , B. Lake , Phys. Rev. B 2018, 98, 214517.

[47] M. Mandal , C. Patra , A. Kataria , D. Singh , P. Biswas , J. Lord , A. Hillier , R. Singh , Phys. Rev. B 2021, 104, 054509.

[48] A. Kataria , Arushi, S. Sharma , T. Agarwal , M. Pula , J. Beare , S. Yoon , Y. Cai , K. Kojima , G. Luke , R. P. Singh , Phys. Rev. B 2023, 107, 174517.

[49] D. Tay , T. Shang , P. F. S. Rosa , F. B. Santos , J. D. Thompson , Z. Fisk , H. R. Ott , T. Shiroka , Phys. Rev. B 2023, 107, 064507.

[50] S. K. Ghosh , M. Smidman , T. Shang , J. F. Annett , A. D. Hillier , J. Quintanilla , H. Yuan , J. Phys. Condens. Matter 2020, 33, 033001. 10.1088/1361-648X/abaa06 32721940

[51] A. D. Hillier , J. Quintanilla , B. Mazidian , J. F. Annett , R. Cywinski , Phys. Rev. Lett. 2012, 109, 097001. 23002872 10.1103/PhysRevLett.109.097001

[52] S. Sumita , Y. Yanase , Phys. Rev. B 2018, 97, 134512.

[53] P. B. Allen , R. C. Dynes , Phys. Rev. B 1975, 12, 905.

[54] W. L. McMillan , Phys. Rev. 1968, 167, 331.

[55] P. B. Allen , Phys. Rev. B 1972, 6, 2577.

[56] P. Morel , P. W. Anderson , Phys. Rev. 1962, 125, 1263.

[57] N. S. Mehta , B. Patra , M. Garg , G. Mohmad , M. Monish , P. Bhardwaj , P. K. Meena , K. Motla , R. P. Singh , B. Singh , G. Sheet , Phys. Rev. B 2024, 109, L241104.

[58] X. P. Yang , Y. Zhong , S. Mardanya , T. A. Cochran , R. Chapai , A. Mine , J. Zhang , J. Sánchez‐Barriga , Z.‐J. Cheng , O. J. Clark , J.‐X. Yin , J. Blawat , G. Cheng , I. Belopolski , T. Nagashima , S. Najafzadeh , S. Gao , N. Yao , A. Bansil , R. Jin , T.‐R. Chang , S. Shin , K. Okazaki , M. Z. Hasan , Phys. Rev. Lett. 2023, 130, 046402. 36763428 10.1103/PhysRevLett.130.046402

[59] K. Shiozaki , M. Sato , K. Gomi , Phys. Rev. B 2016, 93, 195413.

[60] S. Sumita , T. Nomoto , K. Shiozaki , Y. Yanase , Phys. Rev. B 2019, 99, 134513.

[61] H. Chen , J. Gao , L. Chen , G. Wang , H. Li , Y. Wang , J. Liu , J. Wang , D. Geng , Q. Zhang , J. Sheng , F. Ye , T. Qian , L. Chen , H. Weng , J. Ma , X. Chen , Adv. Mater. 2022, 34, 2110664. 10.1002/adma.202110664 35680130

[62] M. Eschrig , Rep. Prog. Phys. 2015, 78, 104501. 26397456 10.1088/0034-4885/78/10/104501

[63] F. Parhizgar , A. M. Black‐Schaffer , npj Quant. Mater. 2020, 5, 42.

[64] J. Cayao , M. Sato , Phys. Rev. B 2024, 110, L201403.

[65] P.‐X. Shen , Z. Lu , J. L. Lado , M. Trif , Phys. Rev. Lett. 2024, 133, 086301. 39241705 10.1103/PhysRevLett.133.086301

[66] A. A. Burkov , M. D. Hook , L. Balents , Phys. Rev. B 2011, 84, 235126.

[67] K. Mullen , B. Uchoa , D. T. Glatzhofer , Phys. Rev. Lett. 2015, 115, 026403. 26207488 10.1103/PhysRevLett.115.026403

[68] R. Yu , Z. Fang , X. Dai , H. Weng , Front. Phys. 2017, 12, 1.

[69] J.‐W. Rhim , Y. B. Kim , Phys. Rev. B 2015, 92, 045126.

[70] S. Ahn , E. J. Mele , H. Min , Phys. Rev. Lett. 2017, 119, 147402. 29053330 10.1103/PhysRevLett.119.147402

[71] Y. Liu , S. A. Yang , F. Zhang , Phys. Rev. B 2018, 97, 035153.

[72] R. Stewart , P. K. Meena , C. Patra , R. K. Kushwaha , T. Agarwal , A. D. Hillier , R. P. Singh 2023, 10.5286/ISIS.E.RB2310137-1.

