# Supplementary material for: Superconductivity in Hourglass Dirac Chain Metals (Ti, Hf)IrGe
Source: Adv Sci (Weinh). 2025 Sep 30;12(43):e12434. doi: 10.1002/advs.202512434 (PMC12631931; doi:10.1002/advs.202512434)
Supplement: Supplementary file 1 — Supporting Information [file ADVS-12-e12434-s001.pdf]

Supplementary material to
"Superconductivity in hourglass Dirac chain metals (Ti, Hf)IrGe"

Pavan Kumar Meena[1,*] Dibyendu Samanta[2,*] Sonika Jangid,[1] Roshan Kumar Kushwaha,[1] Rhea Stewart,[3] Adrian D. Hillier,[3] Sudeep Kumar Ghosh[2,†] and Ravi Prakash Singh[1,‡]

[1]*Department of Physics, Indian Institute of Science Education and Research Bhopal, Bhopal, 462066, India*
[2]*Department of Physics, Indian Institute of Technology, Kanpur 208016, India*
[3]*ISIS Facility, STFC Rutherford Appleton Laboratory, Didcot OX11 0QX, United Kingdom*

In the supplementary material, we present additional details of the synthesis, physical characteristics, data analysis and band structure of the MIrGe (M = Ti, Hf) compounds.

I. BAND STRUCTURE AND TOPOLOGY

We conducted ab initio electronic structure calculations using density functional theory (DFT) as implemented in QUANTUM ESPRESSO. The exchange-correlation effects were treated within the generalized gradient approximation (GGA) using the Perdew-Burke-Ernzerhof (PBE) functional. Electron-ion interactions were described by projector augmented wave (PAW) pseudopotentials. The plane-wave basis set was expanded up to a kinetic energy cutoff of 80 Ry. Brillouin zone integration employed a Γ-centered $8 \times 10 \times 8$ Monkhorst-Pack k-point mesh for bulk calculations. We used experimental lattice parameters and atomic positions derived from Rietveld refinement of X-ray diffraction data.

Figure S1 presents the orbital-resolved projected density of states (PDOS) calculated without spin-orbit coupling (SOC) for MIrGe (M = Ti, Hf). In TiIrGe [Figure S1a], the electronic states near the Fermi level are dominated by Ti-3d orbitals, followed by significant contributions from Ir-5d and Ge-4p orbitals, with minor Ir-5p involvement. This substantial d-orbital character, particularly from Ti-3d and Ir-5d states, elucidates the observed SOC-induced band splitting in TiIrGe. For HfIrGe [Figure S1b], the near-Fermi level states are primarily composed of Hf-5d orbitals, with notable contributions from Ir-5d, Ge-4p, Ir-5p, and Hf-5p orbitals, in descending order of significance. The pronounced presence of 5d orbitals from the heavy elements Hf and Ir accounts for the significant band splitting observed in the SOC-inclusive band structure of HfIrGe.

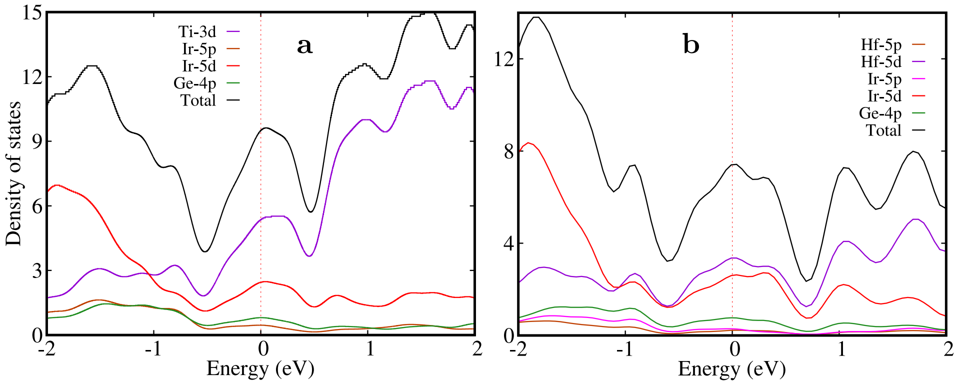

Figure S1. Orbital resolved electronic density of states without spin-orbit coupling (SOC) for a) TiIrGe and b) HfIrGe.

To compute the Fermi surfaces of MIrGe (M = Ti, Hf), we constructed a tight-binding Hamiltonian based on Maximally Localized Wannier Functions (MLWFs). The MLWFs were generated using the WANNIER90 software package. Figure S2 illustrates these Fermi surfaces, obtained without incorporating SOC. Both TiIrGe and HfIrGe exhibit four Fermi surface sheets each, as illustrated in Figure S2a-d and e-h, respectively, with each sheet representing a band that crosses the Fermi level. All Fermi sheets contribute equally to the electronic density of states, underscoring the multi-band nature of both compounds.

To identify the nodal loop, we employed the WANNIER TOOLS package to compute the local energy gap between the two crossing bands. Our result reveals a symmetry-protected nodal ring encircling the Γ point within the $k_x = 0$ plane when SOC is neglected. Figures S3a,b illustrate the nodal loops of TiIrGe and HfIrGe, respectively. This topological feature is preserved by the glide mirror symmetry $\mathsf{G}_x : (x, y, z) \rightarrow (-x + \frac{1}{2}, y + \frac{1}{2}, z + \frac{1}{2})$.

Figure S4 shows the hourglass-type dispersion of TiIrGe along the S–R path, with eigenvalues of G_x indicated. At the S point $(\pi, \pi, 0)$, each Bloch state $|\phi_n\rangle$ forms a degenerate quartet together with its time-reversal $\mathcal{T}|\phi_n\rangle$, parity

* These authors contributed equally to this work
† skghosh@iitk.ac.in
‡ rpsingh@iiserb.ac.in

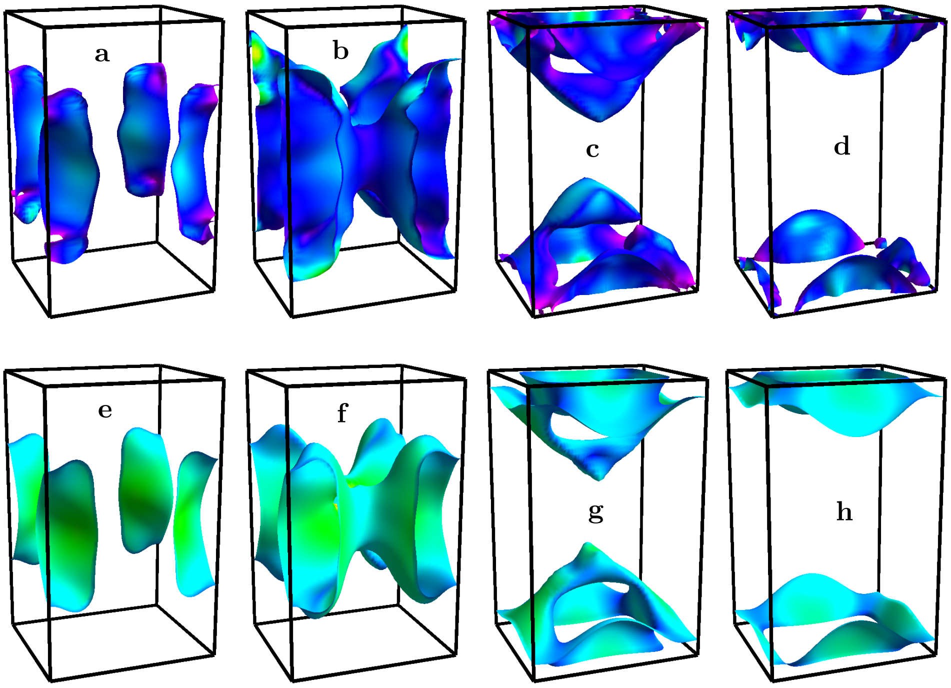

Figure S2. Fermi surfaces without spin-orbit coupling: a-d) TiIrGe and e-h) HfIrGe, illustrating distinct electron and hole pockets in the first Brillouin zone.

$\mathcal{P}|\phi_n\rangle$, and Kramer's $\mathcal{PT}|\phi_n\rangle$ partners. The glide mirror eigenvalues at S are $g_x = \pm 1$, with the $g_x = -1$ states lying at a lower energy than those with $g_x = +1$. At the R point (π, π, π), the glide mirror eigenvalues change to $g_x = \pm i$, meaning that each Kramers pair has opposite eigenvalues. This results in a quartet, with two states having $g_x = +i$ and the other two $g_x = -i$. As the bands evolve from S to R, the redistribution of eigenvalues is enforced by symmetry, inevitably leading to a band crossing. This symmetry-protected crossing gives rise to the characteristic hourglass dispersion shown in Figure S4.
The MIrGe compounds (M= Ti, Hf) lack a global band gap across the Brillouin zone with spin-orbit coupling (SOC). However, the topological invariant $\mathbb{Z}_2$ remains well-defined on the $k_y = 0$ plane. Our calculations reveal that MIrGe compounds have a topological index of $\mathbb{Z}_2 = 1$, indicating nontrivial surface states on the (010) surface. Figure S5a clearly shows the topological surface states crossing the Fermi level for HfIrGe for example. Moreover, these states exhibit a helical spin texture as shown in Figure S5b.

II. CHARACTERIZATION OF MIrGe SAMPLES

Structural characterization: Room temperature powder X-ray diffraction (XRD) measurements of polycrystalline MIrGe (M = Ti, Hf) samples were carried out using a PANalytical X'pert Pro diffractometer machine equipped with CuK_α radiation (λ = 1.5406 Å). Rietveld refinement of the powder XRD patterns, conducted using FullProf Suite software [1], confirm that the samples crystallized in the orthorhombic TiNiSi crystal structure. The refined XRD patterns are shown in Figure S6a,d for TiIrGe and HfIrGe. The obtained lattice parameters are a = 6.2668(9), b = 3.9444(9), and c = 7.3618(4) Å and V$_{cell}$ = 181.98(3) Å^3 for TiIrGe, and a = 6.4902(3), b = 4.0136(8), and c

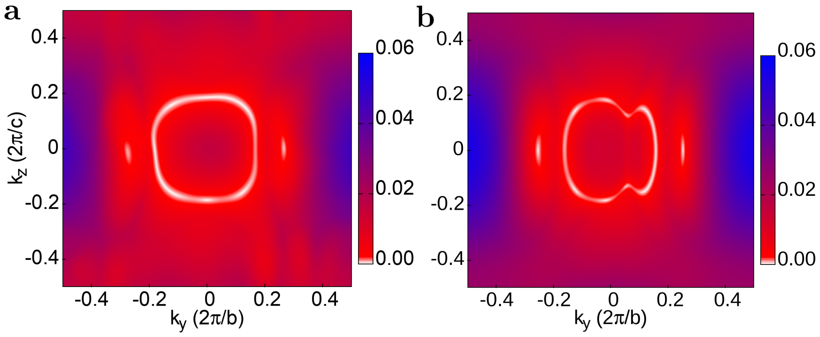

Figure S3. The nodal ring centered at the Γ point in the $k_x = 0$ plane for a) TiIrGe and b) HfIrGe, calculated without SOC.

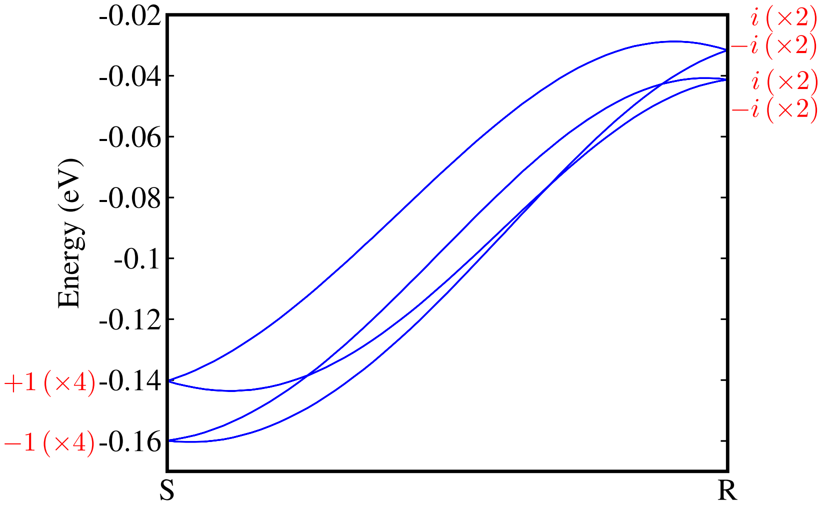

Figure S4. Hourglass-type band dispersion of TiIrGe along the S-R path in the presence of spin-orbit coupling (SOC). The numbers and signs in the figure represent the eigenvalues of the glide mirror symmetry operator, G_x.

Table SI. Structural parameters of MIrGe (M = Ti and Hf) obtained from Rietveld Refinement of powder XRD patterns.

Parameters	TiIrGe	HfIrGe
a (Å)	6.2668(9)	6.4902(3)
b (Å)	3.9444(9)	4.0136(8)
c (Å)	7.3618(4)	7.5196(7)
V_{cell} (Å^3)	181.98(3)	195.88(5)

Atom	Wyckoff position	x	y	z
Ti/Hf	4c	0.027	0.25	0.675
Ir	4c	0.148	0.25	0.064
Ge	4c	0.258	0.25	0.376

= 7.5196(7) Å and V_{cell} = 195.88(5) Å^3 for HfIrGe. These values are consistent with the previous report [2], as detailed with Wyckoff position in Table SI. The elemental composition determined by the EDS spectrum as shown in Figure S6b,e for both polycrystalline compounds suggests a nominal composition.

Electrical resistivity: The temperature-dependent resistivity $\rho(T)$ shown in Figure S6c,f for TiIrGe and HfIrGe compounds exhibit metallic behavior. The obtained residual resistivity ratios (RRR = ρ_{300K}/ρ_{10K}) of 5.14(6) and 6.52(7) for TiIrGe and HfIrGe, respectively, indicate high sample quality. A sudden drop in $\rho(T)$ at a transition temperature T_c of 2.24(5) K for TiIrGe and 5.64(4) K for HfIrGe confirms superconductivity. In addition to the zero-

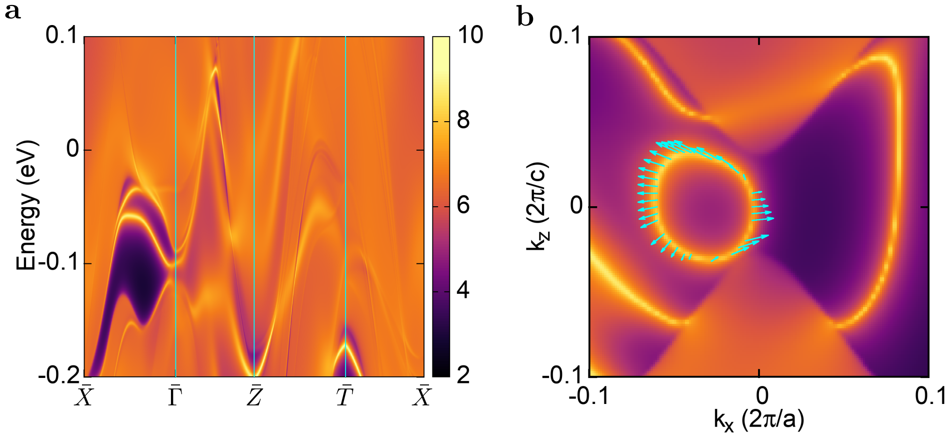

Figure S5. a) Surface state spectrum along high-symmetry paths in the projected (010) 2D surface Brillouin zone of HfIrGe. b) Constant energy contour of the spectrum at -0.090 eV for HfIrGe, with cyan arrows indicating the directions of spins.

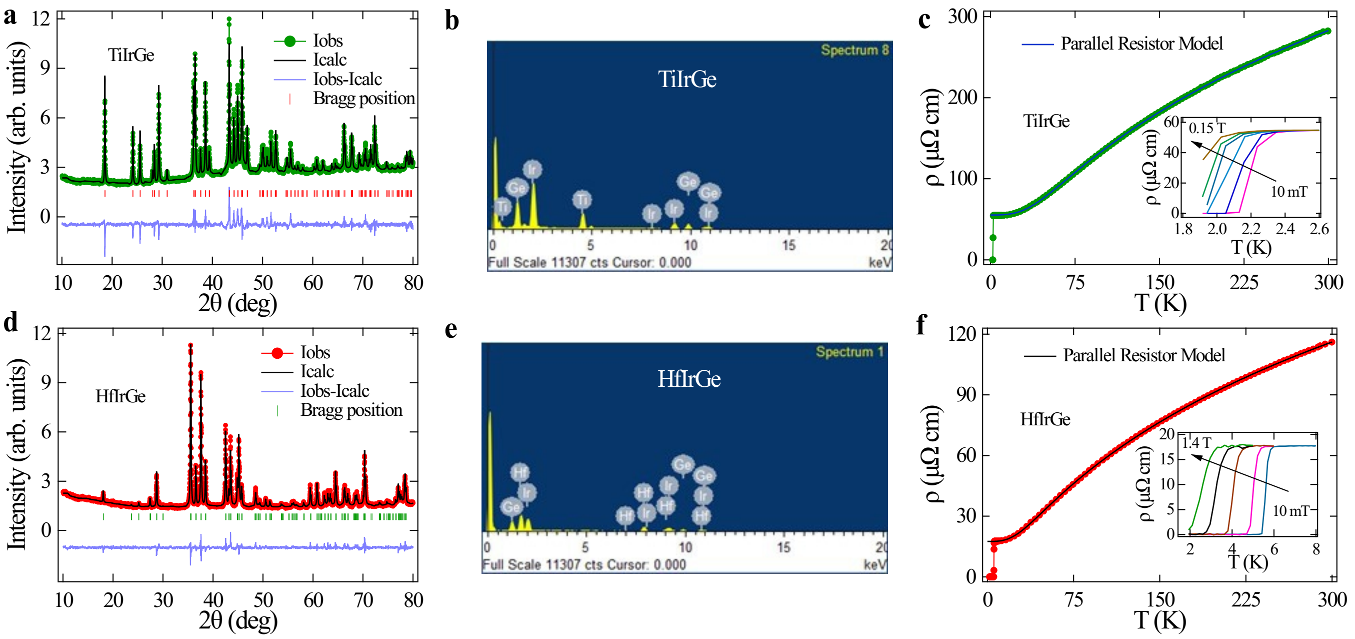

Figure S6. a,d) Rietveld-refined powder XRD patterns for the TiIrGe and HfIrGe compounds, in which round markers represent experimental data, while the black lines correspond to the calculated fit. Vertical bars and lines at the bottom denote Bragg's reflection positions and the difference between observed and calculated intensities for both compounds. b,e) Energy-dispersive X-ray spectroscopy (EDAX) spectra for TiIrGe and HfIrGe compounds, respectively. c,f) Temperature-dependent electrical resistivity measured in zero magnetic field, fitted using the parallel resistor model in the normal state, with insets showing resistivity behavior under various applied magnetic fields.

field measurements, resistivity measurements at different applied magnetic fields, as shown in the inset of Figure S6c,f for TiIrGe and HfIrGe were also performed to evaluate the upper critical field, discussed in the next section. Normal state $\rho(T)$ behavior is well-fitted with the theoretical Wiesmann parallel resistivity model given as [3];

$$\frac{1}{\rho(T)} = \frac{1}{\rho_s} + \frac{1}{\rho_i(T)}, \tag{1}$$

where ρ_s denotes the temperature-independent saturated resistivity corresponding to a mean free path of the order of the interatomic spacing [4]. According to Matthiessen's rule, the ideal contribution to resistivity $\rho_i(T)$ can be written as the sum of two terms: $\rho_i = \rho_{i,0} + \rho_{i,L}$. Here, $\rho_{i,0}$ represents the residual resistivity measured due to impurity scattering, and $\rho_{i,L}$ represents a temperature-dependent contribution of resistivity caused by thermally excited phonons, which can be written as,

$$\rho_{i,L}(T) = C\left(\frac{T}{\Theta_D}\right)^p \int_0^{\Theta_D/T} \frac{x^p}{(e^x-1)(1-e^{-x})}dx, \tag{2}$$

here, Θ_D signifies the Debye temperature, and C is a material-dependent constant. The exponent p can vary depending on the interaction type and is typically set to 3 according to Wilson's theory and 5 as per the BG formula discussed in ref. [5]. The best fit of the data for $p = 3$ (Wilson's theory) yielded a Debye temperature $\Theta_R = 261(5)$ K, $\rho_0 = 59.7(7)$ $\mu\Omega$-cm and $\rho_{sat} = 733.4(2)$ $\mu\Omega$-cm for TiIrGe and $\Theta_R = 184(9)$ K, $\rho_0 = 18.7(2)$ $\mu\Omega$-cm and $\rho_{sat} = 272.4(6)$ $\mu\Omega$-cm for HfIrGe compound, respectively, which are comparable to the structurally similar MIrSi compounds [6].

Magnetization: Further superconductivity was confirmed through magnetic susceptibility and found to be diamagnetic at the superconducting transition temperature for both compounds. A weaker diamagnetic signal for field-cooled cooling (FCC) compared to zero-field cooled warming (ZFCW), suggests flux pinning and reveals type-II superconductivity (see main paper). To calculate the lower critical field $H_{c1}(0)$, the magnetic field-dependent magnetization $M(H)$ at various temperatures in the superconducting state was measured, as shown in the inset of Figure S7a,d for TiIrGe and HfIrGe compounds. The deviation of the $M(H)$ curve from the Meissner linear line at low fields identifies H_{c1} for each temperature. The Ginzburg-Landau (GL) relation $H_{c1}(T) = H_{c1}(0)[1-(T/T_c)^2]$ best fits the extracted temperature dependent H_{c1} data as shown in the Figure S7a,d yielding $H_{c1}(0)$ values of 5.6(1) and 36.4(1) mT. The high H_{c1} values of HfIrGe compared to the TiIrGe may be influenced by grain boundaries in the polycrystalline samples. In addition, the upper critical field $H_{c2}(0)$ is extracted from temperature-dependent magnetization and resistivity measurements under varying magnetic fields. Increasing the magnetic field reduces T_c in the inset of Figure S7b,e (similarly in the resistivity curve). The extracted $H_{c2}(T)$ in Figure S7b,e were best fitted with the GL relation $H_{c2}(T) = H_{c2}(0)\left[\frac{(1-t^2)}{(1+t^2)}\right]$ where $t = \frac{T}{T_c}$, providing $H_{c2}(0)$ as 0.68(1) and 1.36(1) T from magnetization and 0.71(1) and 2.04(1) T from resistivity measurements for TiIrGe and HfIrGe compounds, respectively.

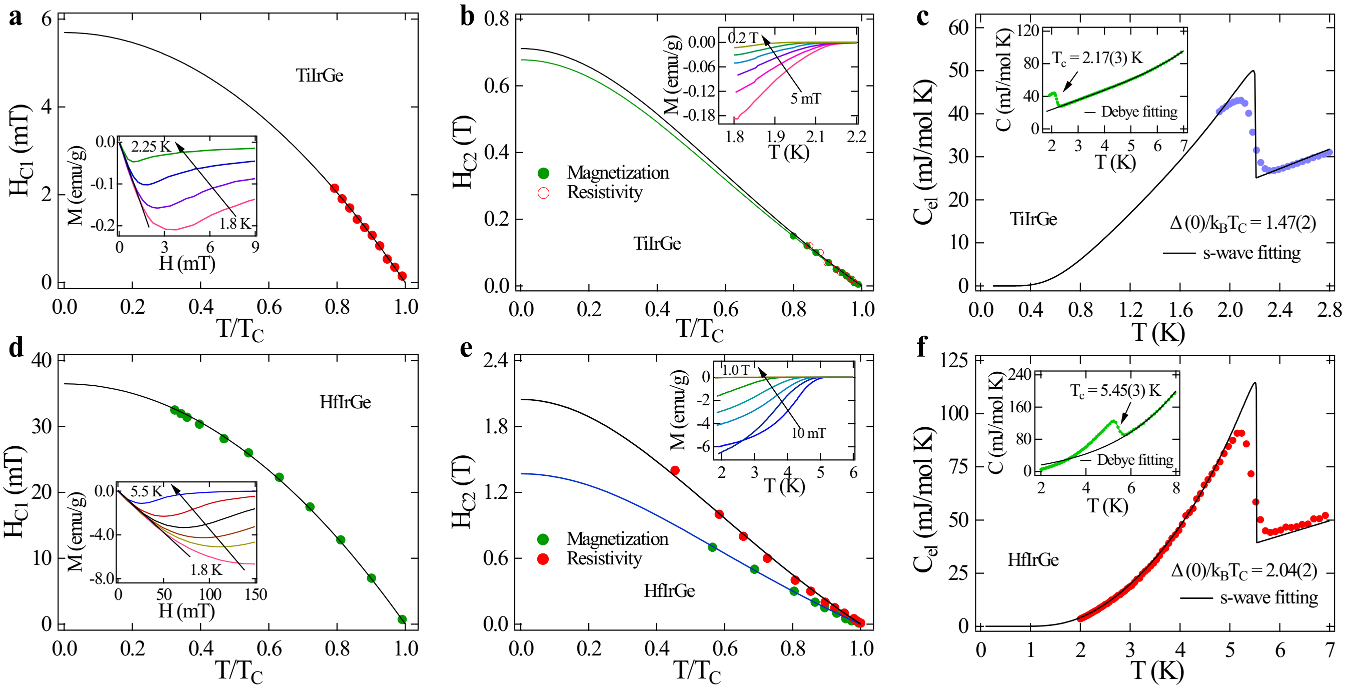

Figure S7. a,d) Temperature dependence of the lower critical field fits with the GL relation, with the inset showing a magnetization curve at different temperatures. b,e) The upper critical field was estimated by fitting the resistivity and magnetization data through the GL relation, with the inset showing magnetization curves at different applied fields. c,f) Temperature-dependent total specific heat fitted using the Debye model for TiIrGe and HfIrGe. respectively.

Superconductivity can be destroyed in two ways under an applied magnetic field: (i) the orbital limiting effect and (ii) the Pauli spin paramagnetic limiting effect. In the orbital limiting effect, the magnetic field increases the kinetic energy of the Cooper pairs, surpassing the condensation energy and leading to the breakdown of superconductivity. For type-II superconductors, the orbital critical field, $H_{c2}^{orb}(0)$, is expressed as $H_{c2}^{orb}(0) = -\alpha T_c \left.\frac{dH_{c2}(T)}{dT}\right|_{T=T_c}$, where α is the purity factor [7, 8]. For $\alpha = 0.693$, the calculated values of $H_{c2}^{orb}(0)$ are 0.53(2) and 1.12(6) T for TiIrGe and HfIrGe compounds, respectively. The Pauli paramagnetic limiting, $H_{c2}^{P}(0)$ field disrupts singlet pairing by aligning electron spins with the applied field. The expression is given as $H_{c2}^{P}(0) = 1.86\ T_c$ [9, 10], resulting in values of 4.17(5) and 10.49(7) T for the compounds TiIrGe and HfIrGe, respectively (T_c used by magnetization). The relative significance of these two limiting mechanisms in suppressing superconductivity is quantified by the Maki parameter, $\alpha_m = \sqrt{2}H_{c2}^{orb}/H_{c2}^{P}$ [11]. The calculated α_m values are 0.18(1) and 0.15(1) for TiIrGe and HfIrGe compounds, respectively, indicating a minor influence of the Pauli limiting effect in these materials.

Consequently, the coherence length $\xi_{GL}(0)$ calculated using the GL formula $H_{c2}(0) = \frac{\Phi_0}{2\pi\xi_{GL}^2}$ (where $\Phi_0 = 2.07 \times 10^{-15}$ Wb is the flux quantum), yielding values of 22.0(2) and 15.5(6) nm (from magnetization) and 21.5(4) and 12.7(2) nm (from resistivity) for TiIrGe and HfIrGe [12]. The superconducting penetration depth $\lambda_{GL}(0)$ is obtained from $H_{c1}(0)$ and $\xi_{GL}(0)$ using the relation: $H_{c1}(0) = \frac{\Phi_0}{4\pi\lambda_{GL}^2(0)}\left[ln\frac{\lambda_{GL}(0)}{\xi_{GL}(0)} + 0.12\right]$, resulting in $\lambda_{GL}(0)$ values of 279.1(5) and 92.6(3) nm for TiIrGe and HfIrGe, respectively. Correspondingly, the GL parameters $\kappa_{GL} = \lambda_{GL}(0)/\xi_{GL}(0)$ are 12.6(8) and 5.9(5) for TiIrGe and HfIrGe compounds, indicating they are strong type-II superconductors, as κ_{GL} exceeds $1/\sqrt{2}$. Using these parameters and the relation $H_c^2.ln\kappa_{GL} = H_{c1}H_{c2}$, the thermodynamics critical magnetic field value H_c of 38.7(3) and 166.6(2) mT was estimated for TiIrGe and HfIrGe, respectively.

Specific heat: The temperature-dependent specific heat measured in a zero magnetic field confirms bulk superconductivity in both compounds, with a superconducting jump observed at $T_c = 2.17(5)$ K and 5.45(3) K for TiIrGe and HfIrGe, respectively, as shown in the inset of Figure S7c,f. The T_c values are consistent with values from other measurements. The normal state data was fitted with the Debye-Sommerfeld relation $C = \gamma_n T + \beta_3 T^3$, where $\gamma_n T$ is an electronic contribution and $\beta_3 T^3$ indicates the lattice contributions. The best fit to the experimental data provides the Sommerfeld coefficient $\gamma_n = 9.96(5)$ mJ/mol-K^2, Debye constant $\beta_3 = 0.09(1)$ mJ/mol-K^4 for TiIrGe, and $\gamma_n = 7.44(3)$ mJ/mol-K^2 and $\beta_3 = 0.21(3)$ mJ/mol-K^4 for HfIrGe. The Debye temperature, θ_D, was calculated using the relation, $\theta_D = \left(\frac{12\pi^4 RN}{5\beta_3}\right)^{1/3}$, where R = 8.314 J mol^{-1} K^{-1} is the gas constant and N = 3 is the number of atoms per formula unit in MIrGe (M = Ti, Hf). From this relation, θ_D was determined to be 399(5) K for TiIrGe and 301(2) K for HfIrGe. Furthermore, the density of state at the Fermi level $D_C(E_F)$ for TiIrGe and HfIrGe compounds are calculated as 4.21(6) and 3.15(6) states eV^{-1} $f.u.^{-1}$ using the relation $\gamma_n = \left(\frac{\pi^2 k_B^2}{3}\right) D_C(E_F)$, where $k_B = 1.38 \times$

10^{-23} J K^{-1}. The electron-phonon coupling, λ_{e-ph}, is then calculated from McMillan's equation [13];

$$\lambda_{e-ph} = \frac{1.04 + \mu^* \ln(\theta_D/1.45T_c)}{(1 - 0.62\mu^*)\ln(\theta_D/1.45T_c) - 1.04}; \tag{3}$$

here, μ^* represents the repulsive screened Coulomb interaction (ranging from 0.07 to 0.15) and is set to 0.13 (for intermetallic compounds). Based on the estimated θ_D and observed T_c values, the calculated λ_{e-ph} values are 0.48(9) and 0.65(7) for TiIrGe and HfIrGe compounds, suggesting weak coupling superconductivity.
Temperature-dependent electronic specific heat $C_{el}(T)$ is obtained by subtracting the phononic part from the total specific heat, which can be related to the entropy S via the relation: $C_{el} = t\frac{dS}{dt}$. The entropy S for a single-gap BCS superconductor can be expressed as [14],

$$\frac{S}{\gamma_n T_c} = -\frac{6}{\pi^2}\left(\frac{\Delta(0)}{k_B T_C}\right)\int_0^\infty [fln(f) + (1-f)ln(1-f)]\,dy. \tag{4}$$

Here, $f(\xi) = (e^{E(\xi)/k_BT} + 1)^{-1}$ is the Fermi function, whereas the integration variable is $y = \xi/\Delta(0)$. $E(\xi)$ is expressed as $\sqrt{\xi^2 + \Delta^2(t)}$ which represents the energy of normal electrons relative to Fermi energy, and $\Delta(t)$ is the temperature-dependent gap function, where t = T/T_c is the reduced temperature. The gap function in the isotropic s-wave BCS approximation is expressed as $\Delta(t) = \tanh[1.82(1.018((1/t) - 1))^{0.51}]$. The low-temperature $C_{el}(T)$ plot aligns well with the isotropic s-wave model in weak-coupling superconductivity, as shown in the Figure S7c,f providing superconducting gap values $\Delta(0)/k_BT_c$ = 1.47(2) and 2.04(2) for TiIrGe and HfIrGe compounds. A similar family of compounds, such as MIrSi, also exhibits weak coupling superconductivity. However, the fitting for the TiIrGe is not determined accurately due to insufficient data points at low temperatures, which is attributed to the instrument's limitation, extending only up to 1.9 K.

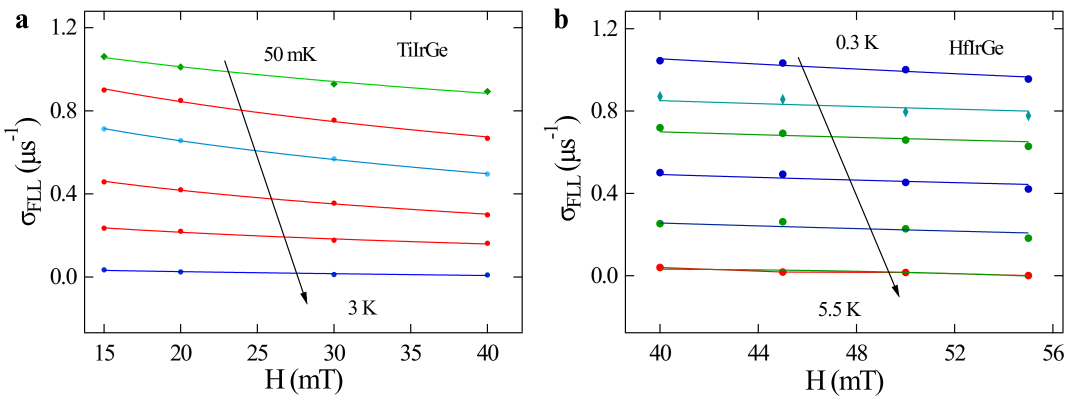

Figure S8. a,b) are the field-dependent relaxation at various temperatures for TiIrGe and HfIrGe compounds, respectively.

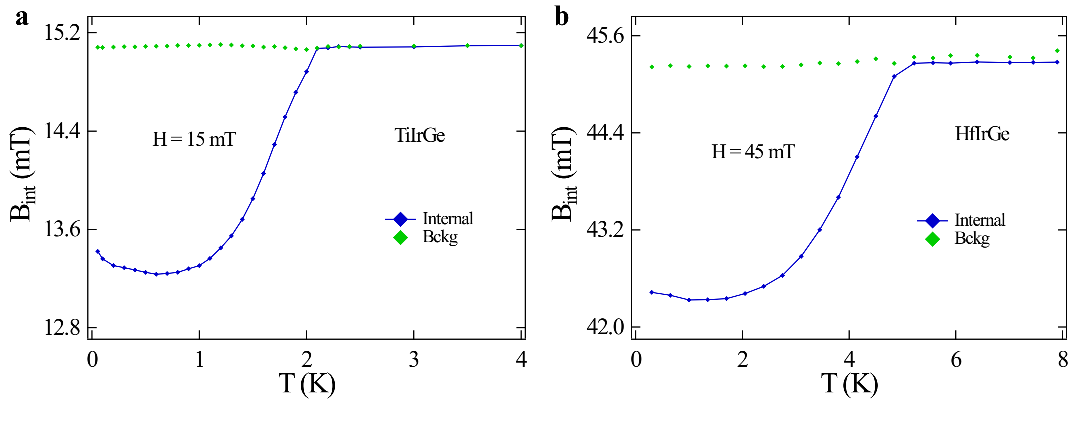

Figure S9. a,b) are the temperature-dependent internal magnetic fields for TiIrGe and HfIrGe compounds, respectively.

III. ANALYSIS OF THE μSR RESULTS

The field-dependent relaxation rate at various temperatures for TiIrGe and HfIrGe compounds are shown in Figure S8a,b. These measurements were used to calculate the penetration depth at low values of the upper critical field for the

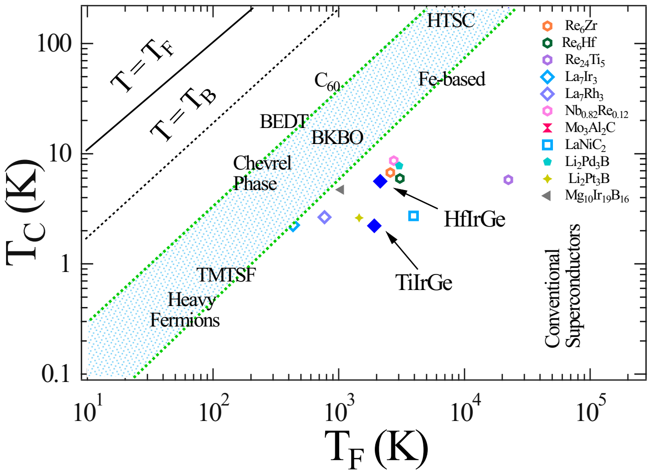

Figure S10. The Uemura plot classifies the superconductor in conventional and unconventional types based on T_C and T_F. MIrGe (M = Ti, Hf) is positioned as a blue marker near the conventional range of superconductivity.

Table SII. Superconducting and normal parameters of MIrGe (M = Ti and Hf) determined from various measurements, including magnetization, resistivity, specific heat, and μSR.

Parameters	Units	TiIrGe	HfIrGe	IrGe [15]
T_c	K	2.24(5)	5.64(4)	4.74
$H_{c1}(0)$	mT	5.6(1)	36.4(1)	13.3(2)
$H_{c2}^{mag}(0)$	T	0.68(1)	1.36(1)	1.13(2)
$H_{c2}^{res}(0)$	T	0.71(1)	2.04(1)	-
H_c	mT	38.7(3)	166.6(2)	78
ξ_{GL}^{mag}	nm	22.0(2)	15.5(6)	17.0
λ_{GL}^{mag}	nm	279.1(5)	92.6(3)	175
$\lambda_{GL}^{\mu SR}$	nm	273.1(6)	246.1(9)	134
k_{GL}		12.6(8)	5.9(5)	10
γ_n	mJ/(mol K^2)	9.96(5)	7.44(4)	3.1
θ_D	K	399(5)	301(2)	160(1)
$\frac{\Delta(0)}{k_B T_C}$ (sp)		1.47(2)	2.04(2)	2.3(1)
$\Delta^{\mu SR}(0)$	meV	0.30(9)	0.75(2)	-
$\frac{\Delta^{\mu SR}(0)}{k_B T_C}$		1.66(7)	1.68(2)	2.1
λ_{e-ph}		0.48(9)	0.65(7)	0.78(2)
$D_C(E_F)$	states/(eV f.u.)	4.21(6)	3.15(6)	-
n	10^{26}	5.6(2)	7.7(3)	-
m^*	m_e	1.48(9)	1.65(7)	-
T_F	K	1921(14)	2136(19)	-

compounds, with the relation and calculated results provided in the main paper. Figures S9a,b show the temperature-dependent internal magnetic field at an applied magnetic field of 15 and 45 mT, for TiIrGe and HfIrGe, respectively. In the superconducting state, the internal magnetic field is less than the applied field due to Meissner field expulsion, but above T_c, it matches the applied field and overlaps with the background magnetic contribution, which is constant over the measured temperature range.

Here, we estimate the superconducting carrier density $n_s = 5.62(4) \times 10^{26}$ and $7.7(3) \times 10^{26}$ m^{-3} by $n_s(0) = m^*/\mu_0 e^2 \lambda^2$, where $m^* = (1 + \lambda_{e-ph}) m_e$. We estimated the Fermi temperature (T_F) for TiIrGe and HfIrGe compounds using the given equation [16], where n and m^* are the electronic carrier density and the effective mass of quasi-particles, respectively:

$$k_B T_F = \frac{\hbar^2}{2} (3\pi^2)^{2/3} \frac{n^{2/3}}{m^*}, \tag{5}$$

The obtained values of T_F are 1921(14) and 2136(19) K, using λ = 2731(6) and 2461(9) Å (from muon spectroscopy measurements) and λ_{e-ph} = 0.48(9) and 0.65(7) (from specific heat data) for TiIrGe and HfIrGe, respectively. The classification of Uemura can divide superconductors as conventional and unconventional based on the ratio of transition temperature and Fermi temperature (T_C/T_F) [17, 18]. If this value comes in the range $0.01 \le \frac{T_C}{T_F} \le 0.1$, then a superconductor is considered an unconventional superconductor; high-T_C superconductors, heavy fermion

superconductors, Fe-based superconductors, as well as Chevrel phase superconductors lie inside this unconventional band on the Uemura plot. The ratios of T_C to T_F are 0.0011(5) and 0.0026(3) by using T_c value of 2.22 and 5.62 K for TiIrGe and HfIrGe compounds, marked by a blue marker in Figure S10 for MIrGe (M = Ti, Hf), indicating its location significantly outside the region of unconventional superconductors.

---

[1] J. Rodríguez-Carvajal, *Physica B.* **1993**, *192*, 1-2 55.
[2] X. Z. Wang, B. Chevalier, J. Etourneau, P. Hagenmuller, *Mater. Res. Bull.* **1987**, *22*, 3 331.
[3] H. Wiesmann, M. Gurvitch, H. Lutz, A. Ghosh, B. Schwarz, M. Strongin, P. B. Allen, J. W. Halley, *Phys. Rev. Lett.* **1977**, *38*, 14 782.
[4] Z. Fisk, G. W. Webb, *Phys. Rev. Lett.* **1976**, *36*, 18 1084.
[5] G. Grimvall, *The electron-phonon interaction in metals*, **1981**.
[6] N. Kase, H. Suzuki, T. Nakano, N. Takeda, *Supercond. Sci. Technol.* **2016**, *29*, 3 035011.
[7] N. R. Werthamer, E. F. Helfand, P. C. Hohenberg, *Phys. Rev.* **1966**, *147*, 1 295.
[8] E. Helfand, N. R. Werthamer, *Phys. Rev.* **1966**, *147*, 1 288.
[9] B. S. Chandrasekhar, *Appl. Phys. Letters* **1962**, *1*.
[10] A. M. Clogston, *Phys. Rev. Lett.* **1962**, *9*, 6 266.
[11] K. Maki, *Phys. Rev.* **1966**, *148*, 1 362.
[12] M. Tinkham, *Introduction to superconductivity*, volume 1, Courier Corporation, **2004**.
[13] W. L. McMillan, *Phys. Rev.* **1968**, *167*, 2 331.
[14] H. Padamsee, J. E. Neighbor, C. A. Shiffman, *J. Low Temp. Phys.* **1973**, *12* 387.
[15] Arushi, K. Motla, P. K. Meena, S. Sharma, D. Singh, P. K. Biswas, A. D. Hillier, R. P. Singh, *Phys. Rev. B* **2022**, *105*, 5 054517.
[16] A. D. Hillier, R. Cywinski, *Appl. Magn. Reson.* **1997**, *13* 95.
[17] Y. J. Uemura, G. M. Luke, B. J. Sternlieb, J. H. Brewer, J. F. Carolan, W. N. Hardy, R. Kadono, J. R. Kempton, R. F. Kiefl, S. R. Kreitzman, et al., *Phys. Rev. Lett.* **1989**, *62*, 19 2317.
[18] Y. J. Uemura, L. P. Le, G. M. Luke, B. J. Sternlieb, W. D. Wu, J. H. Brewer, T. M. Riseman, C. L. Seaman, M. B. Maple, M. Ishikawa, et al., *Phys. Rev. Lett.* **1991**, *66*, 20 2665.
